# Investigating the role of X chromosome breakpoints in premature ovarian failure

**DOI:** 10.1186/1755-8166-5-32

**Published:** 2012-07-16

**Authors:** Simona Baronchelli, Nicoletta Villa, Serena Redaelli, Sara Lissoni, Fabiana Saccheri, Elena Panzeri, Donatella Conconi, Angela Bentivegna, Francesca Crosti, Elena Sala, Francesca Bertola, Anna Marozzi, Antonio Pedicini, Marialuisa Ventruto, Maria Adalgisa Police, Leda Dalprà

**Affiliations:** 1 Department of Neuroscience and Biomedical Technologies, University of Milan-Bicocca, Via Cadore 48, 20900, Monza, MB, Italy; 2 Medical Genetics Laboratory, S. Gerardo Hospital, Via Pergolesi 33, 20900, Monza, MB, Italy; 3 Human Molecular Genetic Consortium, University of Milan-Bicocca, Via Cadore 48, 20900, Monza, MB, Italy; 4 Department of Biology and Genetics for Medical Sciences, University of Milan, Via Viotti 3/5, 20133, Milan, Italy; 5 Medical Genetics Laboratory, S. Giuseppe Moscati Hospital, Viale Italia 1, 83100, Avellino, Italy

**Keywords:** Breakpoint definition, Premature ovarian failure, X chromosome structural aberrations

## Abstract

The importance of the genetic factor in the aetiology of premature ovarian failure (POF) is emphasized by the high percentage of familial cases and X chromosome abnormalities account for 10% of chromosomal aberrations. In this study, we report the detailed analysis of 4 chromosomal abnormalities involving the X chromosome and associated with POF that were detected during a screening of 269 affected women. Conventional and molecular cytogenetics were valuable tools for locating the breakpoint regions and thus the following karyotypes were defined: 46,X,der(X)t(X;19)(p21.1;q13.42)mat, 46,X,t(X;2)(q21.33;q14.3)dn, 46,X,der(X)t(X;Y)(q26.2;q11.223)mat and 46,X,t(X;13)(q13.3;q31)dn. A bioinformatic analysis of the breakpoint regions identified putative candidate genes for ovarian failure near the breakpoint regions on the X chromosome or on autosomes that were involved in the translocation event. HS6ST1, HS6ST2 and MATER genes were identified and their functions and a literature review revealed an interesting connection to the POF phenotype. Moreover, the 19q13.32 locus is associated with the age of onset of the natural menopause. These results support the position effect of the breakpoint on flanking genes, and cytogenetic techniques, in combination with bioinformatic analysis, may help to improve what is known about this puzzling disorder and its diagnostic potential.

## Background

Female infertility is an important health and social disorder and one of its causes is premature ovarian failure (POF, OMIM 311360) which is becoming an increasingly appealing research subject due to its high incidence rate and the absence of an effective treatment [1]. POF is defined as an early ovarian dysfunction characterized by amenorrhea and elevated gonadotropin serum levels before the age of 40 [2]. The median age of natural menopause is around 50, but 9.7% of women experience menopause before 45 (early menopause) and 1.9% under 40 years of age [3].

The pathogenetic mechanisms leading to POF are complex and heterogeneous; the causes may be genetic, autoimmune, infectious or iatrogenic, but a large proportion of POF cases still remain idiopathic [4-6]. Chromosomal defects are frequently associated with POF, especially aberrations involving the X chromosome that account for 5-10% of cases [1,7,8], including numerical and structural abnormalities such as deletions, inversion and X;autosome translocations [9,10]. The cytogenetic and molecular investigations of these abnormalities allowed the identification of two critical regions on the long arm of the X chromosome, at Xq13-q21 and Xq26-27 [11-14]. Candidate genes were identified in the breakpoint regions of the X chromosome, but the actual genetic determinants still remain unknown because these hypothetical candidates need to be confirmed by further investigations [15]. However, the mechanisms that underlie POF’s aetiology might be due to factors other than gene interruption. Indeed, the disruption of critical gene-poor regions might influence the expression of flanking genes [16], or X chromosome translocations might activate the meiotic checkpoint during ovarian follicle maturation due to mispairing [14,17].

In this study, we performed a detailed cytogenetic analysis of 4 chromosomal abnormalities involving the X chromosome and associated with POF that were detected during a screening of 269 affected women [8,18,19]. We identified two *de novo* balanced translocations and two unbalanced, maternally inherited, translocations. Molecular cytogenetic techniques in combination with bioinformatic analysis made it possible to define the breakpoint region of each cytogenetic abnormality and to search for candidate genes involved in the pathogenesis of POF.

## Results

### Banding cytogenetics

**Figure 1:**
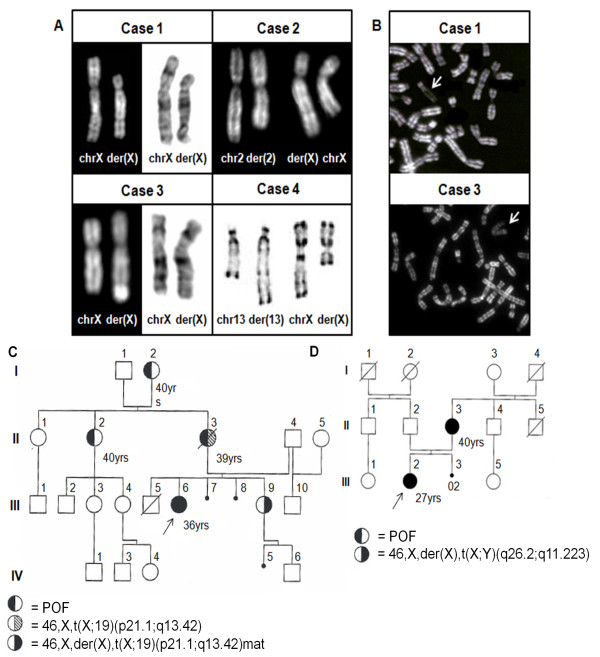
**Conventional cytogenetics.** Partial karyotype of cases 1–4 through QFQ, GTG or RHG banding. **B.** RBA banding allowed the identification of the late replicating derivative X chromosome in cases 1 and 3 (arrows). **C.** Family pedigree of case 1. **D.** Family pedigree of case 3.

Banding cytogenetic techniques, such as QFQ (Q-bands by Fluorescence using Quinacrin), GTG (Giemsa-Trypsin-Giemsa) and RHG (R-bands by heating using Giemsa) banding, showed the presence of abnormal karyotypes in 4 patients and allowed the chromosomal aberrations to be approximately defined (Figure 1A). Chromosome analysis revealed 46,X,der(X)t(X;19)(p21;q13) in case 1;, 46,X,t(X;2)(q21;q14) in case 2; 46,X,der(X)t(X;Y)(q25-26;q11.22) in case 3 and 46,X,t(X;13)(q13.3;q31) in case 4. In case 3 the q arm telomeric region of the derivative chromosome was identified as DA/DAPI (Distamicin A/4',6-diamidino-2-phenylindole) positive (data not shown). Cases 1 and 3 showed maternal inherited chromosomal aberrations. In particular, the mother of case 1 had an X;19 balanced translocation and the sister’s karyotype was the same as the proband. Both were able to conceive, the mother experienced menopause at 39 years of age and the sister had a normal ovarian cycle at the time of diagnosis (Figure 1C). The karyotype of the mother of case 3 revealed the same karyotype as her daughter and she experienced menopause at 40 years of age, a borderline case between POF and early menopause (Figure 1D).

The X inactivation pattern was analysed by means of RBA (Reverse-bands with acridine orange staining) banding for cases 1 and 3 as, in these cases, a derivative chromosome was identified (Figure 1B). In case 1 the derivative X chromosome was late replicating, and so was inactivated, but der(X) resulted completely inactivated in 73% of metaphases, while 27% showed an incomplete and discontinuous inactivation of autosomal material. Case 3 showed a complete late replication of the derivative X chromosome in 100% of the metaphases.

### Molecular cytogenetics and breakpoint mapping

**Figure 2:**
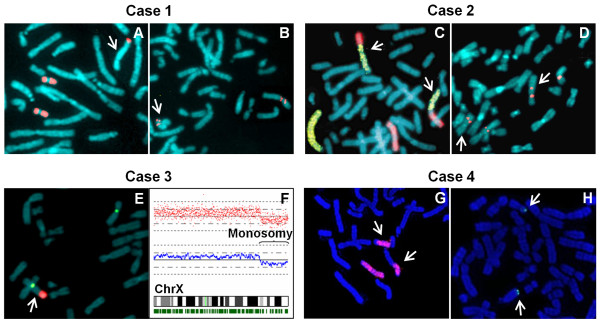
**Molecular cytogenetic analysis. Case 1. A**. FISH analysis by means of wcp19 showing two normal chromosomes 19 and one derivative chromosome positive for wcp19 probe signal. **B.** FISH using RP11-87M18 (Xp21.1) and DXZ1 probes showing hybridization signals both on normal and derivative X chromosomes. **Case 2. C.** Dual colour FISH with wcpX (red) and wcp2 (green) probes showing a X;2 translocation. **D.** FISH using DXZ1 and RP11-150O15 probe, which is present on both derivative chromosomes and on the normal chromosome 2. **Case 3. E.** Dual colour FISH by means of DXZ1 (green) and DYZ1 (red) that identify the heterochromatic Y region on the derivative X chromosome. **F.** SNP analysis localized the breakpoint region in Xq26.2, identified as a monosomy trait from Xq26.2→Xqtel. **Case 4. G.** wcpX probe shows the presence of a translocation between chromosome X and chromosome 13. **H.** 13qtel probe displays hybridization signals on normal chromosome 13 and on derivative X chromosome.

**Figure 3:**
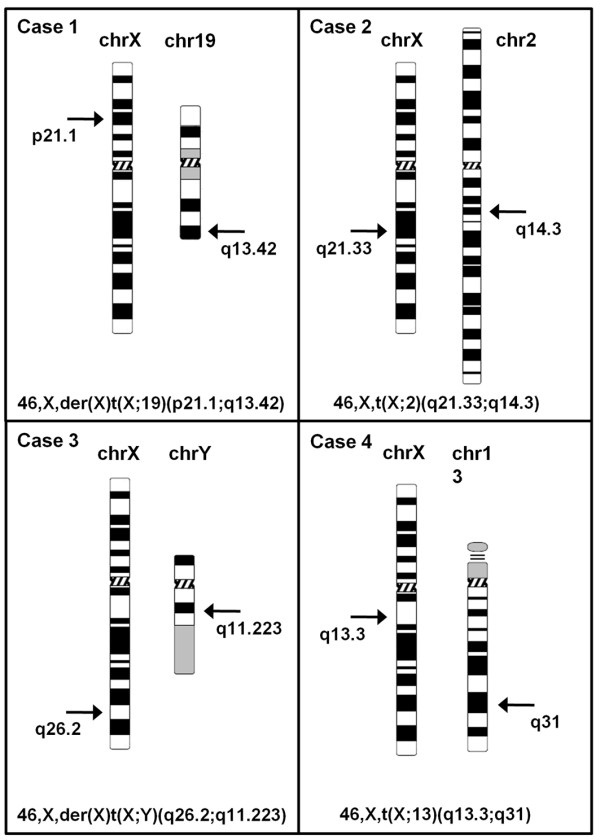
**Breakpoint definitions.** Ideograms showing each specific chromosomal aberration found in cases 1–4 and breakpoint localization

As each chromosomal abnormality is unique, the methodologies used were selected based on case-specific requirements. In order to further characterize the chromosomal abnormalities several FISH (Fluorescence *in situ* hybridization) experiments were assessed by means of different probes corresponding to specific telomeric sequences and partial or whole chromosome libraries (Figure 2). Several FISH experiments using a panel of BAC (bacterial artificial chromosome) and PAC (P1 derived artificial chromosome) probes were carried out in order to determine the exact localization of each chromosomal breakpoint. All the probes used to identify the breakpoints and the results of each hybridization signal are listed in Additional file 1: Table S1. In case 1 the FISH analysis using a wcp19 (whole chromosome painting) probe revealed a derivative chromosome with chromosome 19 material at the telomere of the derivative chromosome q arm (Figure 2A). The breakpoint on chromosome X was localized at Xp21.1 between the RP13-172P16 and RP11-87M18 probes (Figure 2B). Furthermore, the breakpoint on chromosome 19 was mapped at 19q13.42, between RP11-174J9 and CTD-2594I19. A dual colour FISH of case 2, using wcpX and wcp2 probes, confirmed the banding cytogenetic results and revealed an apparently balanced translocation between the long arm of chromosome X and chromosome 2 (Figure 2C). The case 2 breakpoint on the X chromosome was located at Xq21.33 as the RP11-390F10 probe showed the presence of hybridization signals on both the derivative chromosomes (Figure 2D). The RP11-150O15 probe defined the breakpoint on chromosome 2q14.3 for the same reason. In case 3 the DXZ1 and DYZ1 probes allowed the identification of a derivative X chromosome with the presence of Y heterochromatic regions at the telomere of the q arm (Figure 2E). The breakpoint on chromosome X of case 3 was identified by means of a single nucleotide polymorphism (SNP) array analysis (Figure 2F). The association of a loss of heterozygosity (LOH) and a copy number change score of 0.8-1.7 allowed the identification of the breakpoint at Xq26.2, specifically between rs5977559 and rs202735. No other alterations in copy number changes were found in the genome-wide analysis. In order to map the breakpoint on the Y chromosome, we analysed the amplification of microsatellite polymorphic markers included in the Y Chromosome Azoospermia Factor (AZF) Analysis System (Promega, Madison, WI, USA) [Additional file 2. This analysis allowed the breakpoint to be mapped specifically at Yq11.223, between DY5379 and DYF51S1 markers. Case 4 karyotype was characterized by a translocation that occurred between chromosome X and chromosome 13: this hypothesis was confirmed by a FISH analysis using wcpX and 13qtel probes (Figure 2G and 2H). An array comparative genomic hybridization (aCGH) analysis was performed to check whether the translocation was balanced and to reveal any additional alterations. Unfortunately for us, the aCGH data were not informative enough for the breakpoint to be determined as the chromosomal alteration was confirmed as a balanced translocation between chromosome X and chromosome 13. However, cryptic deletions and duplications due to the translocation event were excluded. The aCGH analysis showed 22 copy number variations (CNVs): 14 losses and 8 gains. The CNVs ranged in size from 12Kb to 1270Kb and 16 of them contained known genes [details in Additional file 3: Table S3]. All the observed CNVs overlapped with previously described CNVs: some of them are described as polymorphic on the Database of Genomic Variants [20], others are included in an Italian Database of Human CNVs collected in patients with mental retardation [21] and in none of our patients was any mental retardation noticed. The results of each specific chromosomal aberration, the breakpoint position and the related karyotype are summarized in Figure 3.

### Bioinformatic analysis

A bioinformatic analysis was performed in order to identify possible candidate genes for the POF phenotype. The investigation was performed 1 Mb upstream and downstream from each specific breakpoint, consulting the UCSC Genome Browser [22] and NCBI database [23]. Candidate genes were selected depending on their function, tissue expression and related scientific literature. In case 1 only an open reading frame (cXorf30) and a pseudogene (RPS15AP40) were found in the breakpoint region on chromosome X and no proper genes were detected in the flanking regions. On chromosome 19 the breakpoint fell in 19q13.42. This region, in or near the BRSK gene (+9.8 Kb), has been mapped as being possibly associated with the age at natural menopause [24-26]. Moreover, another interesting gene, MATER (Maternal Antigen that Embryos Require), was found in the trisomic region of chromosome 19 (19q13.42→19qtel). MATER expression is restricted to the oocyte and its activity is essential for early embryonic development [27,28]. In case 2 the breakpoint on X chromosome fell near the DIAPH2 gene (−680Kb). On chromosome 2 the HS6ST1 gene was considered for this analysis. In case 3, in the breakpoint on chromosome X, no genes were found, but the HS6ST2 gene was localized 720Kb downstream. The definition of the Case 4 breakpoint did not help in the identification of candidate genes, suggesting that different mechanisms underlie POF aetiology.

## Discussions

POF is a puzzling disorder as its aetiology is very heterogeneous and most cases are still idiopathic. However, the incidence of familial cases among POF patients is estimated to exceed 30% [29,30], suggesting a genetic basis for some cases of idiopathic POF. In particular, the association between POF and X chromosome abnormalities has been extensively described [4]. Chromosomal anomalies occur in 8.8-33% of women with POF [31] and 10-15% of cases are X chromosome abnormalities, such as numerical and structural aberrations (deletions, inversions and X;autosome translocations) [1,7,8]. This study characterizes the chromosomal abnormalities identified in four patients affected by POF and included in a cohort of 269 patients [8,18,19]. Specifically, we identified 4 chromosomal abnormalities involving the X chromosome with 4 different breakpoint localizations: two *de novo* balanced translocations 46,X,t(X;2)(q21.33;q14.3) in case 2 and 46,X,t(X;13)(q13.3;q31) in case 4 and two maternal inherited unbalanced translocations, 46,X,der(X)t(X;19)(p21.1;q13.42) in case 1 and 46,X,der(X)t(X;Y)(q26.2;q11.223) in case 3. This genetic heterogeneity highlights both the importance of the X chromosome in POF aetiology and the complexity of the POF disorder. However, it is not possible to exclude the involvement of autosomal genes in the onset of ovarian insufficiency as some genes located on autosomes have been associated with the POF phenotype, for example INHA, FSHR and FOXL2 [32-34]. Theoretically, fertility impairment in patients with chromosomal abnormalities might be explained in various ways. First of all, chromosomal anomalies might disrupt a gene that is important for gonadal function [14]; alternatively the breakpoint may fall in gene-poor regions and, in this case, the translocation might induce a long-range position effect in the expression of genes flanking the breakpoint, suggesting an epigenetic control [16,35]. Moreover, structural rearrangements involving the X chromosome may disrupt the normal pairing at meiosis, leading to meiotic arrest [17]. However, the pattern of chromosomal aberrations is still not clearly comprehensible: chromosomal alterations with breakpoints spanning on chromosome X have also been identified in females with normal ovarian function [16,36].

In case 1 the bioinformatic analysis of genes in the breakpoint region on chromosome X did not identify any candidate gene. However, haploinsufficiency for the ZFX gene (X-linked zinc finger protein at Xp21.2) may be an important factor as it has been identified as a candidate gene for ovarian failure [36,37]. The gene content analysis on chromosome 19 raised some interesting points for discussion. Firstly, the breakpoint fell in 19q13.42 and this locus has been mapped as being associated with the age of natural menopause by two independent research groups through genome-wide association studies using SNP analysis [24,26]. In case 1 the breakpoint fell near the BRSK1 gene that might influence the secretion of gonadotropin releasing hormone (GnRH) affecting the menstrual cycle since it is highly expressed in the human brain and is associated with vescicle transport and release at the axonal terminals [24]. Additionally, case 1 might experience partial trisomy for the additional material of chromosome 19 (19q13.42 →19qter) on the derivative X chromosome due to incomplete inactivation of the derivative chromosome [38,39]. Also the MATER gene (Maternal Antigen that Embryos Require) was mapped at 19q13.43 and it is a maternal oocyte protein essential for early embryonic development in mice and an autoantigen associated with autoimmune oophoritis, a mouse model of autoimmune POF [27]. The MATER was identified as a causative gene in a POF patient with a psudic(1;19)(q10;q13.42) by Northup and coworkers [40]. Moreover, the *Mater* gene in mice is specifically transcribed in oocytes [28] and human and mouse MATER genes are conserved and share several structural similarities. Although the exact mechanism of action of the MATER gene product is still unknown, knockout mice show female infertility [27] and so a change in gene dosage (in this case trisomy) might influence fertility [40]. Considering that we have described the second patient as having a chromosomal aberration that involves a putative role for MATER, this gene may be a real candidate in POF aetiology and further investigations may be helpful.

Two isoforms of HS6ST gene were identified in 2 chromosomal breakpoints in our patients: HS6ST1 gene at 2q21 (−216Kb) in case 2 and HS6ST2 gene at Xq26.2 (+720Kb) in case 3. HS6ST1 and HS6ST2 are members of the heparan sulfate sulfotransferase gene family that catalyse the transfer of sulfate to heparan sulfate. Heparan sulfate proteoglicans are ubiquitously expressed on the cell surface and interact with various ligands influencing cell growth, differentiation, adhesion and migration [41]. In 2000 Davison and Conway identified HS6ST as a possible candidate gene for POF aetiology by analyzing the breakpoint on the X chromosome in a family with POF [42]. HS6ST2 in particular is expressed preferentially in the ovary [43] and it might influence oocyte development by inhibiting a proper interaction with follicular growth factors [42]. Moreover, HS6ST1 gene mutations have recently been associated with idiopathic hypogonadotrophic hypogonadism [44], thereby increasing the evidence for a possible role of these two isoforms in gonadal fertility. Taking into account that HS6ST2 was identified as a putative gene responsible for POF phenotype in a family by Davison and co-workers [42], and that we identified the possible involvement of both isoforms in two more cases, the suggestion that this gene family may play a role in POF aetiology is reasonable and should indeed be further investigated.

Additionally, in case 2 the breakpoint fell in Xq21.33, near the DIAPH2 gene (−680Kb), a well-known gene in POF aetiology [45]. The breakpoint was quite distant, but it was not possible to exclude the disruption of some regulatory element *in cis*, or a long-range effect, that would influence gene expression [16].

The case 4 karyotype revealed the breakpoint as being located at Xq13.3 in the POF2 critical region [14,16], but no candidate genes were identified. However, the mechanism underlying the POF phenotype could be due to a direct effect of the chromosomal rearrangement itself without the involvement of specific genes, suggesting a sort of epigenetic control of gene-poor critical regions in patients with X chromosome aberrations [35,46].

The X chromosome inactivation (XCI) pattern is another important feature in unbalanced translocations involving X chromosome. In case 3 RBA banding revealed the complete and preferential inactivation of der(X), whereas in case 1, 73% of metaphases showed a complete inactivation of derivative X chromosome, but 27% of metaphases evidenced an incomplete and discontinuous inactivation of autosomal material, leading to a mosaic for a partial trisomy of chromosome 19 [39]. Furthermore, 15% of genes on the X chromosome escape X inactivation [47] and so the translocation might have caused an improper inactivation of derivative X chromosome and haploinsufficiency of genes involved in ovarian function [19,38]. In case 3 the presence of Y heterochromatic regions may affect the inactivation of der(X) [1,48]. Considering the breakpoint localizations, the XIST (X inactivation-specific transcript) region (Xq13.2) is located on the specific derivative X chromosome of each case. Cases 2 and 4 are balanced translocations and the patients showed no phenotypic abnormality except for ovarian disfunction. Thus, we may assume that XCI in these two cases was skewed, with the derivative X chromosome typically remaining active and the normal X chromosome being inactivated [49]. Indeed, atypical XCI would result in monosomy of autosomal genes, probably leading to a more severe phenotype [50,51].

Cases 1 and 3 are both maternal inherited translocations but the respective mothers experienced menopause at a later age than the daughters. The difference in the age of onset could have several causes. Different X inactivation patterns may influence the age of menopause onset [13,19,52], but also the effect of the genetic background, such as predisposing polymorphisms in the affected individuals, plays a crucial role. In these cases, identical aberrations might cause no apparent symptoms in mothers but severe clinical presentations in the offspring [53-55]. In addition, environmental factors influence the phenotype and, consequently, also the age of menopause onset [56].

aCGH was performed on case 4 and the data analysis revealed no major chromosomal alterations. All the observed CNVs overlapped with described polymorphic CNVs. The comparison with the literature data [49,57-59] showed 2 CNVs significantly associated with the POF phenotype that overlapped with 2 CNVs found in the case 4 molecular karyotype: Xq13.3 [58] and 14q32.33 [59]. However, in our patient these overlapping CNVs were smaller and did not include genes. Moreover, 3 other CNVs described by Aboura and co-workers overlapped with the case 4 CNVs, but these variants were described as not statistically significant compared to CNVs in control populations: 1p36.13, 8p23.1 and 15q11.2 [59]. Although a partial overlapping was found, further studies are required to asses whether there really is an association between these CNVs and the POF phenotype.

## Conclusions

This study confirms the importance of the X chromosome in POF aetiology, but also highlights the complexity of this disorder since different cytogenetic abnormalities lead to the same phenotype. The identification of genes probably involved in ovarian development in the regions flanking the breakpoints supports the hypothesis that the position effect is one of the main mechanisms contributing to the POF phenotype. Detailed cytogenetic definitions of new cases of POF will be instrumental in acquiring further knowledge and in identifying all the genetic determinants involved in the POF aetiology. The link between cytogenetic investigation and bioinformatic analysis may be useful for identifying those putative genes most likely to be involved in ovarian insufficiency.

## Methods

### Clinical population

The cohort investigated in this study has been described previously [8]. Inclusion criteria were the cessation of menses for a period of 6 months or longer before or at the age of 40 and FSH (follicle-stimulating hormone) levels ≥ of 40 IU/l, detected on two different occasions. All of the patients underwent a complete clinical assessment in order to exclude any of the known POF related conditions (i.e. ovarian surgery, autoimmune diseases, Turnerian phenotype). All patients gave their informed consent prior to their inclusion in the study. In this study, we decided to investigate the role of the X chromosome in POF aetiology and, specifically, we characterized the localization of the breakpoint regions in four cases of translocation involving the X chromosome. The age of POF onset was 36 years in case 1, 20 years in case 2, 27 years in case 3 and 22 years in case 4.

### Cell lines

Epstein-Barr virus (EBV)-transformed lymphoblastoid cell lines were provided by the Galliera Genetic Bank, Galliera Hospital (Genoa, Italy). Lymphoblastoid cell lines were grown in RPMI 1640 (Euroclone S.p.A., Milan, Italy) supplemented with 10% foetal bovine serum (PBI International, Milan, Italy) and 2 mM L-glutamine (Euroclone S.p.A., Milan, Italy). Metaphase chromosomes were prepared as previously described [18].

### Banding cytogenetics

Metaphase-chromosome spreads were obtained from phytohaemagglutinin-stimulated peripheral blood lymphocytes. QFQ, GTG, RHG, RBA banding and DA/DAPI staining were performed using standard protocols. At least 20 metaphases were analysed for each sample and a further 50 cells were assessed to exclude sex chromosome mosaicism. The karyotype was expressed following the guidelines of the International System for Chromosome Nomenclature 2009 (ISCN 2009) [60].

### FISH analysis

Fluorescence in situ hybridization was carried out using various commercial probes: wcpX, 13qtel (Vysis, Abbott Molecular, Abbott Park, Illinois, U.S.A.), wcp2, wcp19, DXZ1 and DYZ1 (ONCOR, Gaithersburg, MD, USA). Every experiment was performed according to the manufacturer's instructions. To define the breakpoint regions, probes were selected by consulting the UCSC Genome Browser [22] and NCBI [23]. Bacterial artificial chromosome (BAC) and P1 derived artificial chromosome (PAC) probes were provided by the Wellcome Trust Sanger Institute, UK, and by Prof. M. Rocchi, University of Bari, Italy and are summarized in Additional file 1: Table S1. FISH was performed as described previously [18]. Briefly, probes were labelled by nick translation using digoxigenin(DIG)-11-dUTP (Roche Diagnostics, Indianapolis, IN, USA). Probes were hybridized to metaphase chromosomes overnight and then washed. Detection of digoxigenin probes was obtained by means of 1 μg/ml anti-DIG Rodaminate antibodies (Roche Diagnostics, Indianapolis, IN, USA). Chromosomes were counterstained with DAPI (4',6-diamidino-2-phenylindole). A mean number of 10 metaphases were analysed for each FISH experiment, searching for the presence/absence of probe signals on normal and derivative chromosomes. All the images were taken through a Leica DM 5000B microscope (Leica Microsystems, Wetzlar, Germany) equipped with a Charge Coupled Device (CCD) camera and analysed by means of Chromowin software (Tesi Imaging Srl, Venezia, Italy). The breakpoint localization was determined by FISH analysis and the searching for putative candidate genes in or near the breakpoint region was performed using the most recent human reference sequence (NCBI Build 37.2).

### DNA extraction

Genomic DNA was extracted from patients’ blood or from lymphoblastoid cell-line cultures using the Wizard Genomic DNA purification Kit from Promega according to manufacturer’s protocol and DNA concentration was determined by means of a NanoDrop ND-1000 spectrophotometer (NanoDrop Technologies, Wilmington, DE, USA).

### Microsatellite analysis

The Y Chromosome AZF Analysis System (Promega, Madison, WI, USA) was used for an initial definition of the breakpoint region. Additional microsatellites located on the Y chromosome were used in order to better localize the breakpoint site [Additional file 2]. Microsatellite markers selected for this study showed more than 70% of heterozygosity and they were selected by consulting the UCSC Genome Browser [22].

### Array CGH

A CNV analysis was performed using the Agilent Human Genome CGH Microarray 244A kit (Agilent Technologies, Palo Alto, CA, USA) following the manufacturer’s instructions. Hybridization signals were analysed by means of Feature Extraction software (v10.5) and DNA Analytics software (v5.0, Agilent Technologies, Palo Alto, CA, USA). Aberration Detection Method 2 (ADM2) algorithm (threshold 5.0) was used to identify DNA copy number aberrations. We applied a filtering option of a minimum of 3 aberrant consecutive probes [61] and a minimum absolute average log 2 ratio of 0.30. UCSC human genome assembly hg18 was used as a reference and CNVs were identified with a database integrated into the Agilent Genomic Workbench analytic software. Log 2 ratios lower than −0.30 were classified as losses, those greater than 0.3 as gains.

### SNP analysis

The SNP mapping assay was performed by Genopolis (University of Milan-Bicocca, Milan, Italy) using an Affymetrix 10 K SNP mapping array (Affymetrix, Santa Clara, CA, USA) and following the protocol recommended by the manufacturer. The array was scanned and the signal intensity was measured using GCOS (Gene Chip Operating System). Data were analysed using CNAG (Copy Number Analyser for GeneChip) software version 1.0, evaluating DNA copy number and LOH.

## Abbreviations

ADM2, Aberration Detection Method 2; AZF, Azoospermia Factors; aCGH, array Comparative Genomic Hybridization; POF, Premature Ovarian Failure; BAC, Bacterial Artificial Chromosome; CCD, Charge Coupled Device; CEP, Chromosome Enumeration Probe; CNAG, Copy Number Analyser for GeneChip; CNVs, Copy-Number Variations; DA/DAPI, Distamicin A/4',6-diamidino-2-phenylindole; DIG, Digoxigenin; EBV, Epstein-Barr Virus; FISH, Fluorescence in situ hybridization; FSH, Follicle-stimulating hormone; GCOS, Gene Chip Operating System; GnRH, Gonadotropin releasing hormone; GTG, Giemsa-Trypsin-Giemsa; LOH, Loss of heterozygosity; PAC, P1 derived Artificial Chromosome; PCR, Polymerase chain reaction; QFQ, Q-bands by Fluorescence using Quinacrin; RBA, Reverse-bands with acridine orange staining; RHG, R-bands by heating using Giemsa; SNPs, Single nucleotide polymorphisms; WCP, whole chromosome painting; XCI, X chromosome inactivation; XIST, X inactivation-specific transcript.

## Competing interests

The authors declare that they have no competing interests.

## Authors’ contributions

SB carried out the molecular cytogenetic analysis, bioinformatic analysis, interpretation of data and drafted the manuscript. NV contributed critically to the study design and interpretation of data. SR performed the SNP arrays and aCGH analysis. SL, FS, DC, EP, AB, FB, AP, MV participated in the acquisition of data. ES and FC contributed to the selection of the cohort investigated. LD, AM and MAP participated in the study design and coordination, revised the article and gave their final approval of the version to be published. All authors read and approved the final manuscript.

## Supplementary Material

Additional file 1 Table S1.List of BAC and PAC probes used in the study and results of FISH hybridization signal. Click here for file

Additional file 2 Table S2.Microsatellite markers used to identify the breakpoint on chromosome Y. Click here for file

Additional file 3 Table S3.List of CNVs detected by aCGH of case 4. Click here for file
