# Supplementary material for: Investigating the role of X chromosome breakpoints in premature ovarian failure
Source: Mol Cytogenet. 2012 Jul 16;5:32. doi: 10.1186/1755-8166-5-32 (PMC3443441; doi:10.1186/1755-8166-5-32)
Supplement: Additional file 1 — Table S1.List of BAC and PAC probes used in the study and results of FISH hybridization signal. [file 1755-8166-5-32-S1.pdf]

Table S1 - List of BAC and PAC probes used in the study and results of FISH hybridization signal.

	Clones	Accession No	Cytoband	Coordinates (bp)	FISH hybridisation
**Case 1**	RP13-11B7	AL603753.3	Xp21.1	36 066 298-36 202 463	chrX
	RP13-172P16	AL606467.5	Xp21.1	36 202 464-36 338 556	chrX
	RP11-87M18	AL606516.3	Xp21.1	36 338 557-36 427 501	chrX, der(X)
	RP11-15K21	AL627091.8	Xp21.1	37 163 923-37 191 619	chrX, der(X)
	RP11-174J9	AC116342.2	19q13.42	55 764 587- 55 776 321	chr19
	CTD-2594I19	AC116338.3	19q13.42	55 776 322- 55 785 703	chr19, der(X)
	CTD-2105E13	AC020922	19q13.42	55 803 820- 55 937 878	chr19, der(X)
**Case 2**	RP11-29L10	AL355805.20	Xq21.33	97 447 404- 97 608 700	chrX, der(X)
	RP11-390F10	AL357312.8	Xq21.33	97 542 400- 97 729 868	chrX, der(X), der(2)
	RP11-75F12	AL360173.15	Xq21.33	97 729 868- 97 832 790	chrX, der(2)
	RP11-480N9	AC013277.9	2q14.3	129 085 136- 129 181 869	chr2, der(2)
	RP11-150O15	AC020591.7	2q14.3	129 181 870-129 239 492	chr2, der(2), der(X)
	RP11-414K19	AC012451.8	2q14.3	129 239 493-129 425 613	chr2, der(X)
