# Supplementary material for: Investigating the role of X chromosome breakpoints in premature ovarian failure
Source: Mol Cytogenet. 2012 Jul 16;5:32. doi: 10.1186/1755-8166-5-32 (PMC3443441; doi:10.1186/1755-8166-5-32)
Supplement: Additional file 2 — Table S2.Microsatellite markers used to identify the breakpoint on chromosome Y. [file 1755-8166-5-32-S2.pdf]

Table S2 - Microsatellite markers used to identify the breakpoint on chromosome Y.

STS Marker	Cytoband	Position	Amplification
DYS224	Yq11.223	23 555 947- 23 556 406	-
DYF58S1	Yq11.223	23 962 002-23962 371	-
DYS26	Yq11.223	24 164 187-24 364 338<br>24 514 373-24 714 524	-
DYS379	Yq11.223	24 631 231-24 631 480	-
DYS236	Yq11.223	25 223 858-25 423 982<br>25 234 706-25 434 830<br>26 890 393-27 090 517	+
DYF51S1	Yq11.223	25 665 544- 25 665 686	+
