# Supplementary material for: Investigating the role of X chromosome breakpoints in premature ovarian failure
Source: Mol Cytogenet. 2012 Jul 16;5:32. doi: 10.1186/1755-8166-5-32 (PMC3443441; doi:10.1186/1755-8166-5-32)
Supplement: Additional file 3 — Table S3.List of CNVs detected by aCGH of case 4. [file 1755-8166-5-32-S3.pdf]

Table S3 - List of CNVs detected by aCGH of case 4.

Chromosome: nucleotides	Cytoband	Size (Kb)	log2ratio (CN)	Genes
1: 16719747-16964322*	p36.13	244.6	**-0.40(CN=1.52)**	NBPF1, CROCCL1, MSTP2, ESPNP, MSTP9
1: 150823073-150852905	q21.3	29.8	**-1.49(CN=0.71)**	LCE3C, LCE3B
1: 246794322-246875192	q44	80.9	**0.59(CN=3.01)**	OR2T34, OR2T10, OR2T11, OR2T35
5: 771310-826103	p15.33	54.8	**0.52(CN=2.88)**	-
6: 32567382-32601021	p21.32	33.6	**-1.62(CN=0.65)**	HLA-DRB5
6: 79035891-79080047	q14.1	44.2	**-4.92(CN=0.07)**	-
6: 109698258-109729234	q21	31	**-0.65(CN=1.27)**	-
7: 38259147-38385763	p14.1	126.6	**-0.30(CN=1.62)**	TARP
7: 142159154-142171665	q34	12.5	**-1.28(CN=0.82)**	TRY6
8: 7729311-8132183*	p23.1	402.9	**-0.54(CN=1.38)**	DEFB104A, DEFB104B, SPAG11B, SPAG11A, DEFB103B, DEFB103A, DEFB4, FAM66E, DEFB109, FLJ10661, miRNA:hsa-mir-548i-3
8: 39356595-39505315	p11.23-p11.22	148.7	**0.66(CN=3.16)**	ADAM5P, ADAM3A
10: 11220557-11275743	p14	55.2	**-0.33(CN=1.59)**	CUGBP2
12: 9528590-9585215	p13.31	56.6	**0.79(CN=3.46)**	-
13: 102503934-102516734	q33.1	12.8	**-0.39(CN=1.53)**	SLC10A2
14: 18624383-19490689	q11.1-q11.2	866.3	**-0.55(CN=1.37)**	OR4Q3, OR4M1, OR4N2, OR4K2, OR4K5, OR4K1
14: 21381173-22046156	q11.2	665	**-0.23(CN=1.71)**	-
14: 105946993-105994705§	q32.33	47.7	**0.39(CN=2.62)**	-
15: 18810004-20079994*	q11.2	1270	**-0.77(CN=1.17)**	LOC727832, GOLGA8C, LOC646214, CXADRP2, POTEB, LOC727924, OR4M2, OR4N4, LOC650137, miRNA:hsa-mir-1268
17: 41521544-41706929	q21.31	185.4	**0.54(CN=2.91)**	KIAA1267
22: 22671374-22725353	q11.23	54	**0.72(CN=3.29)**	GSTTP1, LOC391322, GSTT1, GSTTP2
22: 37689058-37715431	q13.1	26.4	**-0.49(CN=1.42)**	APOBEC3A, APOBEC3B
X: 75872352-75906023°	q13.3	33.8	**0.24(CN=2.36)**	AK057746

Gain/**Loss**; CN = copy number
*= partially overlapping with nonstatistically significant CNVs described in POF patients [1].
§= partially overlapping with a statistically significant CNV described in POF patients [1].
°= partially overlapping with a CNV described in POF patients [2]

References

1. Aboura A, Dupas C, Tachdjian G, Portnoï MF, Bourcigaux N, Dewailly D, Frydman R, Fauser B, Ronci-Chaix N, Donadille B *et al*: **Array comparative genomic hybridization profiling analysis reveals deoxyribonucleic acid copy number variations associated with premature ovarian failure.** *J Clin Endocrinol Metab* 2009, **94**(11):4540-4546.

2. Quilter CR, Karcanias AC, Bagga MR, Duncan S, Murray A, Conway GS, Sargent CA, Affara NA: **Analysis of X chromosome genomic DNA sequence copy number variation associated with premature ovarian failure (POF).** *Hum Reprod* 2010, **25**(8):2139-2150.
